# Veterinary Practitioners’ Standpoints and Comprehension towards Antimicrobial Use—Are There Opportunities for Antimicrobial Stewardship Improvement?

**DOI:** 10.3390/antibiotics11070867

**Published:** 2022-06-27

**Authors:** Zorana Kovačević, Jovana Vidović, Mihajlo Erdeljan, Marko Cincović, Zoran Ružić, Ivan Galić, Tijana Kukurić, Nenad Stojanac, Olga Horvat

**Affiliations:** 1 Department for Veterinary Medicine, Faculty of Agriculture, University of Novi Sad, 21000 Novi Sad, Serbia; zorana.kovacevic@polj.edu.rs (Z.K.); jovanavidovic21@gmail.com (J.V.); erdeljanm@polj.uns.ac.rs (M.E.); mcincovic@gmail.com (M.C.); ivangalicvet@gmail.com (I.G.); tijana.kukuric@gmail.com (T.K.); stojanac.n@gmail.com (N.S.); 2 Faculty of Medicine, University of Novi Sad, 21000 Novi Sad, Serbia; olgahorvat15@gmail.com

**Keywords:** rational antimicrobial use, prudent antimicrobial use, antimicrobial stewardship, antibiotics, antimicrobial resistance, veterinarians, Serbia

## Abstract

The main subject of the research is the assessment of the knowledge, attitudes and behaviors of veterinarians regarding the use of antibiotics (AMU) and antimicrobial resistance (AMR) through a questionnaire conducted among veterinarians in the northern region of Serbia. A total of 62 respondents completed the questionnaire, which represents a response rate of 44.3%. Male veterinarians are less likely to be in the group of veterinarians with insufficient knowledge (*p* < 0.05). Veterinarians engaged in mixed practice (small and large animals) (*p* < 0.001) and veterinarians who have over 100 patients per month (*p* < 0.005) are also less likely to be in the group with insufficient knowledge of antimicrobial resistance. The proportion of those with insufficient knowledge is growing among veterinarians whose source is the Internet (*p* < 0.01), while the proportion of those with insufficient knowledge about antimicrobial resistance is declining among veterinarians whose source of information is continuous education (*p* < 0.05). The majority of the respondents (*n* = 59, 95.2%) completely agreed that AMR is a very big issue in the global health sector right now. Unfortunately, there are crucial gaps in the knowledge and attitudes of the surveyed participants. They do not appear to be aware of the importance of AMU in veterinary medicine and its influence on overall AMR, or the crucial part that non-prescribed antibiotics have in all of it. Positively, many veterinarians use good practice AMU guidelines in their everyday practice and in line with the global trend of AMU reduction, respondents have also decreased their AMU compared to the previous year.

## 1. Introduction

Antimicrobial resistance (AMR) poses a global public health concern that influences both humans and animals [1,2] and the emergence of AMR limits the possibilities for treating infectious diseases [3,4].

Solutions such as preventive measures, including the enhancement of antimicrobial stewardship (AMS) in humans [5,6] and veterinary practice [7,8], as well as raising awareness and education on the AMR issue among healthcare professionals, have found their place to combat this immense problem. In 2015, the World Health Organization (WHO) set up the Global Action Plan on AMR, where education on the AMR issue among all healthcare professionals is highlighted [9]. In addition, the WHO principles pointed out the importance of training veterinarians on this issue on a graduate and postgraduate level also, as continuing education [10].

Antimicrobial resistance affects both humans and animals and resistance can also spread from animals to humans through the food chain or direct contact [11,12,13]. In the European Medicines Agency (EMA) and European Food Safety Authority (EFSA) Joint Scientific Opinion on antimicrobial use (AMU) in animal husbandry, it was pointed out that due to the multiplicity of factors contributing to AMR, the impact of any single measure is difficult to quantify, although there is evidence of an association between the reduction in AMU and reduced AMR [14]. Indeed, interventions designed to reduce AMU in food-producing animals have a positive effect on reducing the prevalence of AMR in both animals and humans that are in contact with food-producing animals [15]. In fact, AMR is promoted by the indiscriminate use of antibiotics in the veterinary, agricultural, and medical sectors [16].

According to the recently available valid antibiotic-use and antimicrobial resistance data, Serbia belongs to a group of European countries with the highest rates of resistance, as well as with a high antibiotic consumption rate in human medicine [17,18,19,20]. Although the European Surveillance of Veterinary Antimicrobial Consumption (ESVAC) report data on the use of antimicrobial agents in animals from 31 European countries [21], Serbia still is not part of it. In low- and middle-income countries (such as Serbia), AMR poses a particularly significant threat due not only to the health-care challenges these countries face, but also to an increase in small-scale intensive animal production, exacerbated by poor sanitation infrastructure [22]. In 2019, following the resolution of the United Nations by all countries, Serbia formulated its National Antimicrobial Resistance Control Program for 2019–2023, where among other strategies, special attention is given to increasing awareness among those who prescribe antimicrobials [23].

Inappropriate antimicrobial prescribing patterns in human medicine have been investigated all over the world [24,25,26,27], as well as research on the drivers of this use in veterinary medicine [28,29,30,31,32,33]. Awareness of the current guidelines and appropriateness of AMU in veterinary practice was also studied [34]. Hence, although veterinarians’ knowledge on the AMR and AMU issue has been explored in Italy [35], the Netherlands [36] and Nigeria [37], in Serbia, the only thorough study has been conducted among farm animal veterinarians [38], but no one focused on predominantly small animal veterinary practitioners. Furthermore, higher AMR in companion animals follow higher AMU (both companion and food-producing animals), as reported by ESVAC [21].

The identification of factors that influence the decision-making process involved in the selection of antimicrobials by veterinarians is important and could help to develop future strategies and interventions regarding AMU on a postgraduate level and incorporate it in the continuous education of current veterinarians. Hence, the objective of this study was to identify and evaluate the knowledge, attitudes and beliefs towards AMU and AMR among predominantly small animal practice veterinarians in the northern region of Serbia, the South Bačka district.

## 2. Results

### 2.1. Sociodemographic Data

A total of 62 veterinarians successfully completed the questionnaire. More than half (58.1%) of the respondents were male (*n* = 36) and the rest were female (*n* = 26, 41.9%). The majority of respondents were between the ages 30 and 39 years old (*n* = 45, 72.6%) and only a few were between the ages of 40 and 49 years old (*n* = 3, 4.8%). Almost all participants worked in a private practice (*n* = 57, 91.9%) and had a title of DVM (*n* = 60, 96.8%). More than half (*n* = 36, 58.1%) of the surveyed veterinarians had 6–15 years of experience working in practice. A summary of the demographic data is presented in Table 1.

### 2.2. Knowledge of AMR

Almost all (*n* = 59, 95.2%) participants agree that AMR is an important public health problem, with zero veterinarians disagreeing with the statement. More than two thirds (*n* = 46, 74.2%) of the veterinarians think that new antibiotics will be introduced that will resolve the issue of AMR. More than half of the respondents (*n* = 37, 59.7%) also strongly disagree that AMU in veterinary medicine is an important cause of resistance to bacterial infections in humans. Participants also strongly (*n* = 29, 32.3%) or slightly (*n* = 17, 27.4%) agree that prescription antibiotics should be more controlled. More than two thirds of the veterinarians strongly agree (*n* = 42, 67.6%) that AMR is mainly a hospital-related issue. Knowledge of antimicrobial resistance is presented in Table 2.

### 2.3. Significance of AMR in Everyday Practice

Half of the respondents (*n* = 32, 51.6%) have attended some educational programs about the AMU or AMR issue within the last three-year period, while one quarter (*n* = 16, 25.6%) did not attend any at all. Most veterinarians (*n* = 27, 43.5%) encountered resistant bacteria on a monthly basis. The veterinarians that showed insufficient knowledge of AMR consider rational AMU on farms (*n* = 50, 80.6%), rational use of antibiotics by the patient (*n* = 48, 77.4%) and the rational prescribing of antibiotics by a veterinarian (*n* = 42, 67.7%) to be the most important sectors to focus on to slow down the development of AMR. On the other hand, the veterinarians that showed sufficient knowledge of AMR considered the most important sectors to be hygiene in hospitals (*n* = 51, 82.3%), hygiene during food preparation and consumption (*n* = 49, 79%) and hygiene on farms (*n* = 48, 77.4%). Significance of antibiotic resistance in everyday practice is presented in Table 3.

### 2.4. Antibiotic Prescribing Habits

With regard to AMU in comparison to the same period of last year, two-thirds (*n* = 41, 66.1%) of the respondents decreased while a few (*n* = 9, 14.5%) increased the amount of antibiotics they prescribe. Four of the most prescribed antibiotics were amoxicillin and clavulanic acid (*n* = 31, 50.0%), penicillin (*n* = 30, 48.4%), enrofloxacin (*n* = 30, 48.4%) and cephalosporins (*n* = 28, 45.2%). The veterinarians that showed sufficient knowledge of AMR mostly prescribe antibiotics without a clear indication because of linguistic/cultural barriers in communication with animal owners (*n* = 59, 95.2%), in non-compliance with the owner of the animal by the instructions of the veterinarian in taking the medication (*n* = 57, 91.9%) and if the owner of the animal requires an antibiotic (*n* = 55, 88.7%). On the other hand, the veterinarians that showed insufficient knowledge of AMR mostly prescribe antibiotics when there is a lack of clear guidelines for some conditions (*n* = 39, 62.9%), because of the cost of microbiological tests (*n* = 28, 45.2%) and lack of rapid diagnostic tests (*n* = 22, 35.5%). Most respondents routinely conduct antibiotic sensitivity tests (AST or antibiogram) after failure in applied therapy (*n* = 42, 67.7%). Most participants rarely or never (*n* = 38, 61.3%) prescribe antibiotics outside of their registered use indications. Antibiotic prescribing habits is presented in Table 4.

### 2.5. Sources of Information

More than half of the respondents use domestic or foreign guidelines moderately (*n* = 36, 58.1%) when prescribing antibiotics in their daily work; however, almost all of them (*n* = 59, 95.2%) would also like to have more local guidelines available. The respondents that have sufficient knowledge of AMR mostly use internet forums (*n* = 45, 72.6%), good practice guidelines (*n* = 35, 56.5%), textbooks (*n* = 32, 48.4%) and scientific journals (*n* = 32, 48.4%) as up-to-date sources of information for antibiotic therapy and AMR and almost all (*n* = 60, 96.8%) think that there is no need for additional sources as the current ones are adequate. Sources of information are presented in Table 5.

### 2.6. Logistic Regression for Factors Predicting Poor Antibiotic Knowledge as the Dependent Variable

Logistic regression shows that veterinary demographics related to gender, type of practice, and number of patients are significant predictors in recognizing insufficient knowledge about antimicrobial resistance. Male veterinarians are less likely to be in the group of veterinarians with insufficient knowledge (OR = 0.298; 95% CI = 0.092–0.965, *p* < 0.05), veterinarians engaged in mixed practice (small and large animals) (OR = 0.153; 95% CI = 0.068–0.344, *p* < 0.001) and veterinarians who have over 100 patients per month (OR = 0.136; 95% CI = 0.038–0.479, *p* < 0.005) are also less likely to be in the group with insufficient knowledge of antimicrobial resistance.

The veterinarians who answered that hygiene on farms (OR = 0.066;95% CI = 0.018–0.247, *p* < 0.001) and the rational prescribing of antibiotics by veterinarians (OR = 0.232 (95% CI = 0.076–0.712), *p* < 0.05) are significant sectors of work that should be focused on in order to slow down the development of resistance are much less likely to be in the group with insufficient knowledge about antibiotic resistance.

Antibiotic prescribing habits, such as the selection of amoxicillin/clavulanic acid for the first-line antibiotic (OR = 6.171; 95% CI = 1.87–20.362), *p* < 0.005), prescribing antibiotics without a clear indication when the weekend is approaching (OR = 28,235; 95% CI = 3.45–231.1], *p* < 0.005) and because microbiological analyzes are expensive (OR = 20.462; 95% CI = 4.137–101.21), *p* < 0.001), increases the chance that the veterinarian is found in a group with insufficient knowledge of resistance. Routine antibiogram preparation in case of unsuccessful treatment reduces the chance of the veterinarian being in the group with insufficient knowledge (OR = 0.231; 95% CI = 0.074–0.722, *p* < 0.05).

Sources of information on antimicrobial resistance can be important predictors in the detection of veterinarians with insufficient knowledge. The proportion of those with insufficient knowledge is growing among veterinarians whose source is the Internet (OR = 9048 (95% CI = 1863–43,947), *p* < 0.01), while the proportion of those with insufficient knowledge about antimicrobial resistance is declining among veterinarians whose source of information is continuous education (OR = 0.213; 95% CI = 0.054–0.837, *p* < 0.05). Factors predicting poor antibiotic knowledge are presented in Table 6.

## 3. Discussion

As mentioned before, evaluation of the current and future veterinarians’ knowledge regarding AMU is important as it is one of the bases if we want to have any chance of successfully reducing AMR as one of the greatest threats to global health. The only published research so far in Serbia focused solely on the farm animal veterinarians’ knowledge and attitudes toward AMR and AMU [38], as well as veterinary students’ comprehension of these topics [39]. Interestingly, no one has yet questioned all the veterinarian specialists from the one part of the country, such as the South Bačka District in Serbia. With respectable numbers of ambulances in small animal practice and farms, as well as being the second largest teaching base for veterinary education, this district could be a close reflection of the prescribing habits regarding antibiotics among veterinarians in the whole of Serbia.

The majority of the participants in the current study were 30–39 years old, with more than half having 6–15 years of experience working in practice and having attended some educational programs about the AMU or AMR issue within the last three-year period. Most of the respondents work in small (54.8%) or mixed practices (37.1%) that are predominantly small animal practice oriented.

In the current study, the veterinarians that showed sufficient knowledge of AMR considered hygiene in hospitals, hygiene during food preparation and consumption and hygiene on farms to be the most important sectors to focus on to slow down the development of AMR. Interestingly, all these mentioned reasons are part of the antimicrobial stewardship (AMS) principals [40], which have to be adopted in veterinary sectors, both private and public. Furthermore, AMS practices are encouraged among veterinarians to reduce the indiscriminate use of antimicrobials, as well as to improve AMU in animal health care delivery [41].

On the other hand, the veterinarians that showed insufficient knowledge of AMR thought the focus should be on rational AMU in farm animals, the rational use of antibiotics by the animal owner and the rational prescribing of antibiotics by a veterinarian. This is very surprising since there is evidence that veterinarians with sufficient knowledge of AMR can influence the decrease in AMU [42]. Veterinarians in other studies seemed to be highly [42] or moderately [43] aware of how important the reduction in AMU is for the AMR issue. Farmers/animal owners do have a significant role to play in the misuse of antimicrobials [44]; however, studies show that veterinarians have a significant influence on the farmers’ attitudes [45,46]. According to the data reported by Dickson et al. [47], the promotion of appropriate antibiotic stewardship for pet owners and vets may offer a viable pathway for planning interventions, benefitting from synergies with other interventions that target prescribers. Furthermore, it is recommended that veterinarians should be involved in the education of pet owners and farmers on responsible AMU [48]. This puts the focus on the continuous education of the current and future veterinarians on AMR and AMU connections, as well as regarding AMS understanding and consequently improvement.

Two-thirds of the respondents decreased AMU in comparison to the same period last year, while a few (14.5%) increased the amount of antibiotics they prescribe. This is certainly a positive trend, since one of the main goals of the global action to reduce the levels of AMR is the decrease in AMU [8,49], especially since it has been estimated that 73% of the global AMU is in the livestock sector [50].

Four of the most prescribed antibiotics by the surveyed veterinarians were amoxicillin and clavulanic acid (50.0%), penicillin (48.4%), enrofloxacin (48.4%) and cephalosporins (45.2%). All of these antibiotics are considered critically important for human medicine [51,52]. As in the current study, veterinarians all over the Europe frequently prescribe critically and highly important drugs for both human and veterinary medicine as the first therapeutic choice [53,54,55]. This highlights the urgency in reducing AMR through good antimicrobial stewardship [56] and working on finding new antibiotics [57] or alternatives to antibiotics [58]. In addition, prudent AMU has to be promoted in the veterinary sector at all levels, along with limiting the non-judicious usage [59]. Furthermore, it is vital that all countries implement the appropriate systems to ensure that antimicrobials are manufactured, marketed, distributed, prescribed, supplied and used responsibly, and that these systems are adequately audited [60]. Prudent AMU is important since in many cases, clinical cure does not guarantee bacteriological cure. In fact, the success of antimicrobial therapy depends on factors such as achieving penetration of the drug to the infection site in a sufficient concentration (pharmacokinetics) and the potency and efficacy of the drug against infecting microorganisms at the infection site (pharmacodynamics, as well as compliance with the administration of the prescribed drug according to the recommended dosage schedule) [61].

Commendably, most participants in the current study rarely or never prescribe antibiotics outside of their registered use indications. Some research showed that often the determining factors for veterinary professionals when prescribing antibiotics are financially motivated [62,63]. A systematic review article that included 34 studies on the non-clinical factors influencing veterinarians’ prescribing habits suggested that they did not seem to be susceptible to their socio-demographic characteristics; however, they were influenced by different attitudes, namely fear, self-confidence, business factors, and complacency, but also owner-related factors, for example lack of awareness and demand for antibiotics [64]. Mapping veterinarians’ drivers of AMU is essential, since besides clinical signs and bacteriological outcome, the emergence of AMR is one of the assessment criteria for the success (or failure) of metaphylaxis or therapy, with a spectrum of possibilities [61].

With regard to prescribing antibiotics without a clear indication, the veterinarians that showed sufficient knowledge of AMR mostly do it because of linguistic/cultural barriers in communication with the animal owners, in non-compliance with the owner of the animal by the instructions of the veterinarian in taking the medication and if the owner of the animal requires an antibiotic. Although none of the health professionals leveraged antibiotic prescription as a way to gain the trust of pet owners [65], they have been found to have substantial influence over veterinary decision-making on antibiotic use [47].

On the other hand, the veterinarians that showed insufficient knowledge of AMR mostly prescribe antibiotics when there is a lack of clear guidelines for some conditions, because of the cost of microbiological tests and the lack of rapid diagnostic tests. Interestingly, a study conducted in Germany showed that doctors of human medicine (GPs) expressed a need for better guidelines more frequently than veterinarians (GPs 42%, hospital physicians 42%, veterinarians 15% [66]). Furthermore, the irrational use of antimicrobials is certainly a complex and multifactorial problem in developing countries, and a proper understanding of the problem is necessary for effective control policies [67]. Hence, insight into the veterinarians’ habits in prescribing antibiotics could be the first step in the design of educational interventions.

A study that included 25 European countries suggested that the most important factors influencing veterinarians’ selection of an antibiotic in therapy are as follows: antibiotic susceptibility test (AST or antibiogram) results, their own experience, the risk of developing AMR, and ease of administration [68]. In the current study, most respondents routinely conduct antibiotic susceptibility tests (AST) after failure in applied therapy, which is similar to other research results [37,69]. On the other hand, only 15% of the questioned veterinarians in Chile reported the use of laboratory diagnostic tests [70].

Even though AST is not a perfect method and does not guarantee treatment success as there are many factors that influence the efficacy and execution of these methods and therapy [71], it is still a crucial instrument in the veterinary sector for the selection of the most appropriate agent for the treatment of bacterial diseases of animals [72].

Good practice AMU guidelines are one of the most efficient methods of fighting antibiotic misuse/excessive use and by extension, reducing AMR [73]. For this reason, it is important for veterinary professionals to use and follow the available guidelines. In the current study, more than half of the respondents use domestic or foreign guidelines moderately when prescribing antibiotics in their daily work; however, almost all of them would also like to have more local guidelines available. Other studies conducted in the USA have had similar results [74,75].

One of the recommendations for AMU decrease is the continued provision and promotion of guidelines and relevant education to veterinarians at both under- and postgraduate levels, which is necessary to further improve the uptake of responsible AMU and AST [48]. Although AMS guidelines represent an important tool to help veterinarians optimize their AMU with the objective of decreasing AMR, an overview of the available AMU guidelines in small-animal veterinary practices gave us insight into the need for national guidance documents in multiple European countries, as well as in Serbia [76]. This is the reason why scientists all over the world put in effort to improve the existing guidelines and especially, to develop and implement new guidelines.

The respondents that have sufficient knowledge of AMR mostly use Internet forums, good practice guidelines, textbooks and scientific journals as up-to-date sources of information for antibiotic therapy and AMR and almost all think that there is no need for additional sources, as the current ones are adequate. These results are comparable to a USA study, suggesting that veterinarians received information regarding antimicrobials from textbooks/drug handbooks, ongoing professional development courses, peer reviewed scientific literature and pharmaceutical companies [77]. However, the high placement of the Internet forums in the current study is disparaging, as it is related to a higher level of insufficient knowledge of AMR.

Almost all (95.2%) participants agree that AMR is an important public health problem, which corresponds with most Bhutan veterinarians (96%) in similar research [78]. More than two thirds (74.2%) of veterinarians think that new antibiotics will be introduced that will resolve the issue of AMR. Although this is a very positive attitude as the World Health Organization is aiming for AMR reduction by the development of new antibiotics [79], this seems to be a very challenging task, and it is probably easier to develop safe and efficient alternatives to antibiotics [80]. On the other hand, it seems as even though a number of nonantibiotic alternative antimicrobial approaches show interesting potential, it is difficult to understand how they would displace the need for new antibiotics for the foreseeable future [81].

One third of the respondents strongly agrees that AMU in veterinary medicine is an important cause of resistance to bacterial infections in humans. This is in line with a study about veterinary students from Croatia and Serbia that suggested that students are not very aware of veterinary medicine AMU contribution to overall AMR, as only 56.8% have chosen strong contribution as the answer [39]. One third of the participants also strongly or slightly agree that prescription antibiotics should be more controlled. This is a very subpar attitude, since the research suggests that the prevalence of AMR is higher in countries and regions where the use of non-prescription antibiotics is more frequent [82]. Additionally, more than two thirds of veterinarians in the current study strongly agree that AMR is mainly a hospital-related issue. These results are unfavorable and show a significant lack of understanding of the correlations between AMU in the veterinary sector with the possibilities of emergence of AMR in humans.

## 4. Study Limitations

The main limitations of the current study can be related to the sample of veterinarians and the methodology. Questionnaire studies have certain limitations by their very nature, including their subjectivity and reliance on the participants’ opinions, reasoning skills and memory. Misinterpretation and possible ambiguity of questions is also a likelihood that we tried to reduce with as many closed, distinct questions as possible. This questionnaire was conducted in the northern region of Serbia (Southern Bačka) and might not be completely representative of the whole country. A key limitation is that only a small proportion of veterinarians participated, and consequently the majority of the analyses needed to be restricted to veterinarians in the northern region of Serbia. Despite this limitation, we have been able to identify some key knowledge gaps among the veterinarians participating in our study. Furthermore, our pilot methodology, including the survey instrument developed, should be useful for informing future work. Such studies may aim to produce more generalizable results, and would need to target a greater number of participants from different regions in Serbia. Our response rate was 44.3%, which is comparable or higher lever compared to other similar studies [36,83]. Being comparable with other studies in the response rate and also representing a pilot study, this study could be valuable in strategies for AMS improvement for AMR control.

## 5. Materials and Methods

### 5.1. Ethical Approval

The study was approved by the Ethical Committee of the Veterinary Chamber of Serbia (approval number 423/15/12/2020) before the research was conducted.

### 5.2. The Questionnaire

The questionnaire was drafted by the authors in the Serbian language with the intention to assess the attitudes, knowledge and behaviors of practicing veterinarians regarding AMU in Serbia during the period from 14th of March to 14th of September 2020. The questionnaire was created using a combination of original questions and certain modified questions from various surveys [43,68,84]. It was pre-tested on 20 veterinarian colleagues to make sure the questionnaire was clear, unbiased and that its content was easily comprehensible and valuable. The questionnaire was adjusted in accordance to the feedback from the piloted sample; however, the data of the pilot study were not included in the final analysis. Further face-validation, as well as objective validation, for comprehensibility and clarity was conducted via consultation with expert colleagues in the field. The questionnaire was distributed personally to 140 working veterinary practitioners in the region of Southern Bačka (confirmed data via personal communication with the Veterinary Chamber of Serbia, November 2020) in Serbia. Sixty-two veterinarians successfully completed the questionnaire, corresponding to the response rate of 44.3%. The questionnaire should not have taken more than 10 min to be completed. It consisted of 22 questions divided in 5 parts by subjects, which are as follows: sociodemographic data, significance of AMR in everyday practice, antibiotic prescribing habits, sources of information and knowledge of AMR.

The first part consisted of nine questions regarding demographic data, including age, gender, work sector, graduation year, highest educational degree, specialty, type of practice, years working in practice and the monthly average number of treated animals. The second part had three questions focused on the significance and attitudes of veterinarians towards AMR in every day practice. The third part had five questions on the veterinarians’ habits when prescribing antibiotics with the aim to assess their awareness of the possible consequences and responsibility they have. The fourth part included four questions about the sources of information veterinarians potentially use to educate themselves and improve their professional knowledge on these issues. Finally, the fifth part was used to evaluate the respondents’ knowledge of AMR by using seven statements for which they had to choose five different levels of agreement (strongly agree, slightly agree, neither agree or disagree, slightly disagree and strongly disagree).

The whole questionnaire was translated and is available in English as an online Supplementary File S1 (“Questionnaire on the knowledge, attitudes and behavior of veterinarians regarding antimicrobial use and antimicrobial resistance”). Dataset containing responses collected by questionnaire is available in English as an online Supplementary File S2.

### 5.3. Data Analysis

The gathered results were imported to Microsoft Excel and checked for consistency and uniformity errors. For statistical processing, the veterinarians were classified into those who have insufficient knowledge of antimicrobial resistance (marked with 1 and carry <28 points) and veterinarians who have sufficient knowledge of antimicrobial resistance (marked with 0 and carry >28). In Likert’s questionnaire, the answers that were correct are decoded, so they carry 5 points each, which is a pandam to the answer “I strongly agree”. The cut off value was 28 points, because this number of points speaks in favor of the fact that the respondent answered “yes” to all 7 questions on average, which is numbered 4 on the scale, and the product is 28. Univariate logistic regression was used, with the answers from the questionnaire as an independent variable and a dichotomous assessment of the knowledge of antimicrobial resistance as a dependent variable. The results are presented through regression parameter B, statistical significance, odds ratio (OR) with its 95% interval. All the tests conducted were two-tailed. The alpha level of significance for all the inferential statistics was set at 0.05, unless otherwise specified. The statistical analysis was conducted using SPSS software (version 20.0; SPSS Inc, USA).

## 6. Conclusions

The assessed knowledge, attitudes and behaviors of predominantly small animal veterinarians towards AMU highlight very mixed results in regards to these subjects. Very favorably, the veterinarians consider AMR to be a significant health problem; however, many of them consider it mainly a hospital-related issue. They also do not appear to be aware of the importance of AMU in veterinary medicine and its influence on overall AMR, or the crucial part that non-prescribed antibiotics have in all of it. Positively, in line with the global trend of AMU reduction, the respondents have decreased their AMU compared to the previous year and they also rarely prescribe antibiotics outside of their indications. Many veterinarians use good practice AMU guidelines in their everyday practice; however, there is clearly a need for more local guidelines.

Unfortunately, there are crucial gaps in the knowledge and attitudes of the surveyed participants. It seems highly necessary to further push continuous education and the spread of information in the veterinary sector regarding AMU and AMR at both an undergraduate and postgraduate level. Improvement in AMS knowledge seems to be necessary as a part of AMR control in both the human and veterinary sector. Furthermore, similar studies should also be conducted in the future to assess the situation and follow the progress of Serbian veterinarians towards this important issue.

## Figures and Tables

**Table 1:** Sociodemographic data.

Variable	Response	Frequency (*n* = 62)	Percentage (%)
Age	50–60	10	16.1
	40–49	3	4.8
	30–39	45	72.6
	25–29	4	6.5
Gender	Male	36	58.1
	Female	26	41.9
Work sector	State	5	8.1
	Private	57	91.9
Years since graduation	30–40	3	4.8
	20–29	7	11.2
	10–19	22	35.4
	1–9	30	48.4
Title	DVM	60	96.8
	MSc	2	3.2
Specialty	None	41	66.1
	Reproduction and Obstetrics	6	9.7
	Surgery	5	8.1
	Dermatology	2	3.2
	Cattle breeding	2	3.2
	Prevention and therapy of small animals	2	3.2
	Toxicology	2	3.2
	Ultrasound diagnostics	2	3.2
Type of practice	Small and large practice	23	37.1
	Small practice	34	54.8
	Large practice	5	8.1
Number of years in practice	0–5	18	29
	6–15	36	58.1
	>15	8	12.9
Number of monthly cases	0–50	17	27.4
	51–100	14	22.5
	101–500	26	42.0
	501–3000	5	8.0

**Table 2:** Knowledge of antimicrobial resistance (1-strongly disagree; 2-disagree; 3-neither agree nor disagree; 4-agree; 5-strongly agree).

Statements		1.00	2.00	3.00	4.00	5.00
Bacterial resistance to antibiotics is an important public health problem in our environment.	*n*	0	0	1	2	59
	%	0	0	1.6	3.2	95.2
Prescribing antibiotics to patients affects the possible occurrence of bacterial resistance to these drugs.	*n*	0	0	3	9	50
	%	0	0	4.8	14.5	80.6
I am convinced that new antibiotics will be introduced that will solve the problem of resistance.	*n*	6	5	3	2	46
	%	9.7	8.1	4.8	3.2	74.2
The use of antibiotics in animals is an important cause of resistance to bacterial infections in humans.	*n*	37	1	2	1	21
	%	59.7	1.6	3.2	1.6	33.9
The two most important causes of antibiotic resistance are self-medication and antibiotic abuse.	*n*	7	6	11	21	17
	%	11.3	9.7	17.7	33.9	27.4
Prescription antibiotics should be more controlled.	*n*	10	5	10	17	20
	%	16.1	8.1	16.1	27.4	32.3
The problem of antibiotic resistance of bacteria is mainly a problem in hospital settings.	*n*	1	2	8	9	42
	%	1.6	3.2	12.9	14.5	67.7

**Table 3:** Significance of antibiotic resistance in everyday practice.

Question	Response	Frequency (*n* = 62)	Percentage (%)
Have you attended any educational program (seminar, educational meeting, continuing education) on the rational use of antibiotics or antibacterial resistance?	Yes, more than 3 years ago	12	19.4
	Yes, during the last 3 years	32	51.6
	I did not	16	25.8
	I do not remember	2	3.2
How often do you encounter infections caused by bacteria resistant to most antibiotics in your daily work?	Daily	6	9.7
	Monthly	27	43.5
	Weekly	10	16.1
	Rarely or never	19	30.6
What sectors do you think should be focused on in order to slow down the development of antibiotic resistance? (0-Respondents did not choose an answer; 1- Respondents chose the answer)	Hygiene in hospitals	0 51 1 11	82.3 17.7
	Hygiene during food preparation and consumption	0 49 1 13	79.0 21.0
	Hygiene on farms	0 48 1 14	77.4 22.6
	The rational use of antibiotics in hospitals	0 27 1 35	43.5 56.5
	The rational prescribing of antibiotics by a veterinarian	0 20 1 42	32.3 67.7
	The rational use of antibiotics by the animal owner	0 14 1 48	22.6 77.4
	The rational use of antibiotics in farm animals	0 12 1 50	19.4 80.6

**Table 4:** Antibiotic prescribing habits.

Question		Response	*n* = 62	%
Your prescription of antibiotics is in relation to the same period of the previous year.		Decreased	41	66.1
		Increased	9	14.5
		There was no change	12	19.4
List the four antibiotics that you most often prescribe in your daily work.		Amoxicillin and clavulanic acid	31	50.0
		Penicillin	30	48.4
		Enrofloxacin	30	48.4
		Cephalosporins	28	45.2
What are the reasons for prescribing antibiotics without a clear indication? (0-Respondents did not choose an answer; 1- Respondents chose the answer)	When the weekend is approaching and it is difficult to predict the course of the disease	0	51	82.3
		1	11	17.7
	If the owner of the animal requires an antibiotic	0	55	88.7
		1	7	11.3
	Non-compliance with the owner of the animal by the instructions of the veterinarian in taking the medication	0	57	91.9
		1	5	8.1
	Linguistic/cultural barriers in communication with animal owners	0	59	95.2
		1	3	4.8
	Lack of rapid diagnostic tests	0	40	64.5
		1	22	35.5
	Costs of microbiological tests	0	34	54.8
		1	28	45.2
	Lack of clear guidelines for some conditions	0	23	37.1
		1	39	62.9
Do you routinely carry out an antibiogram in case of failure of the applied therapy?		Yes	42	67.7
		No	20	32.3
How often do you prescribe antibiotics because of indications for which they are not approved and registered for use?		Often	5	8.1
		Rarely or never	38	61.3
		Moderately	19	30.6

**Table 5:** Sources of information.

Question		Response	*n* = 62	%
Do you use domestic or foreign guidelines when prescribing antibiotics in your daily work?		Often	20	32.3
		Moderately	36	58.1
		Rarely or never	6	9.7
		There are no good guidelines	0	0
Would you like to have more local guidelines for the rational use of antibiotics?		Yes	59	95.2
		I do not know	3	4.8
		No	0	0
What are your sources for obtaining up-to-date information on antibiotic therapy and antibiotic resistance? (0-Respondents did not choose an answer; 1- Respondents chose the answer)	(a) Internet forums	0	45	72.6
		1	17	27.4
	(b) Textbooks	0	32	51.6
		1	30	48.4
	(c) Scientific journals	0	32	51.6
		1	30	48.4
	(d) Guides to good clinical practice	0	35	56.5
		1	27	43.5
	(e) Direct communication with colleagues	0	21	33.9
		1	41	66.1
	(f) Continuous education	0	19	30.6
		1	43	69.4
What additional sources of information would be particularly useful?	(a) There is no need for additional sources, The existing ones are sufficient	0	60	96.8
		1	2	3.2
	(b) Clear national guidelines	0	28	45.2
		1	34	54.8
	(c) More continuing education that is not sponsored by the pharmaceutical industry	0	11	17.7
		1	51	82.3

**Table 6:** Logistic regression for factors predicting poor antibiotic knowledge as the dependent variable.

Predictive Variable		B	Sig.	OR	95% C.I. for OR	
					Lower	Upper
Sociodemographic data	Year of birth	−0.008	0.811	0.992	0.927	1.062
	Gender, m—1; f—0	−1.212	0.043	0.298	0.092	0.965
	Company, private—1; state—0	22.144	0.999	~	~	~
	Year of graduation	−0.018	0.660	0.983	0.909	1.063
	Highest level of education, DVM—1; MR—0	−20.584	0.999	0.000	0.000	~
	Specialty, yes—1; no—0	0.366	0.529	1.442	0.461	4.511
	Type of practice, small—1; large—2; small and large—3	−1.876	0.000	0.153	0.068	0.344
	Number of years in practice, 0 to 5–1; 6 to 15–2; >15–3	0.180	0.680	1.197	0.509	2.817
	Monthly average number of patients, <100–0. >100–1	−1.997	0.002	0.136	0.038	0.479
Significance of antibiotic resistance in daily work	Have you attended any education on the rational use of antibiotics or antibacterial resistance? I did not—1; Yes, more than 3 years ago—2; Yes, during the last 3 years—3; I do not remember—4	0.043	0.894	1.043	0.559	1.947
	How often do you encounter infections caused by bacteria resistant to most antibiotics in your daily work? Rarely—1; Monthly—2; Weekly—3; Daily—4	0.028	0.920	1.028	0.602	1.756
	What sectors do you think should be focused on in order to slow down the development of antibiotic resistance? On hygiene in hospitals	0.983	0.238	2.672	0.521	13.691
	On hygiene on farms	−2.715	0.000	0.066	0.018	0.247
	On hygiene during food preparation and consumption	−0.254	0.694	0.776	0.219	2.754
	The rational use of antibiotics in hospitals	0.074	0.897	1.077	0.351	3.303
	The rational prescribing of antibiotics by a veterinarian	−1.460	0.011	0.232	0.076	0.712
	Rational antibiotic intake by patients	−0.788	0.271	0.455	0.112	1.851
	The rational use of antibiotics in farm animals	0.417	0.527	1.518	0.417	5.527
Habits in prescribing antibiotics	Your prescription of antibiotics is in relation to the same period of the previous year: Decreased—1; There was no change—2; Increased—3	0.211	0.649	1.235	0.497	3.073
	List the four antibiotics that you most often prescribe in your daily work: 1 Amoxicillin and clavulanic acid	1.820	0.003	6.171	1.870	20.362
	2 Penicillin	0.143	0.800	1.154	0.381	3.493
	3 Enrofloxacin	0.032	0.960	1.032	0.301	3.537
	4 Cephalosporins	0.281	0.649	1.324	0.395	4.435
	What are the reasons for prescribing antibiotics without a clear indication? When the weekend is approaching and it is difficult to predict the course of the disease	3.341	0.002	28.235	3.450	231.1
	If the owner of the animal requires an antibiotic	−0.433	0.596	0.649	0.131	3.211
	Non-compliance with the owner of the animal by the instructions of the veterinarian in taking the medication	−0.288	0.763	0.750	0.115	4.876
	Linguistic/cultural barriers in communication with animal owners	−22.026	0.999	0.000	0.000	~
	Lack of rapid diagnostic tests	0.143	0.800	1.154	0.381	3.493
	Costs of microbiological tests	3.019	0.000	20.462	4.137	101.21
	Lack of clear guidelines for some conditions	0.065	0.907	1.067	0.360	3.160
	Do you routinely do an antibiogram in case of failure of the applied therapy? No—0; Yes—1	−1.466	0.012	0.231	0.074	0.722
	How often do you prescribe antibiotics in indications for which they are not approved and registered for use? Rarely or never—0; Moderately—1; Often—2	−0.752	0.169	0.471	0.161	1.378
Sources of information	Do you use domestic or foreign guidelines when prescribing antibiotics in your daily work? Rarely or never—0; Moderately—1; Often—2	0.206	0.637	1.229	0.522	2.896
	Would you like to have more local guidelines for the rational use of antibiotics? No—0; I do not know—2; Yes—3	−0.025	0.984	0.975	0.083	11.415
	What are your sources for obtaining up-to-date information on antibiotic therapy and antibiotic resistance? Internet forums	2.203	0.006	9.048	1.863	43.947
	Textbooks	0.336	0.534	1.400	0.485	4.038
	Scientific journals	0.047	0.931	1.048	0.366	3.002
	Guides to good clinical practice	0.339	0.536	1.403	0.480	4.106
	Direct communication with colleagues	−0.366	0.529	0.693	0.222	2.168
	Continuous education	−1.547	0.027	0.213	0.054	0.837
	What additional sources of information would be particularly useful? There is no need for additional sources, the existing ones are sufficient	20.584	0.999	~	~	~
	Clear national guidelines	−0.141	0.794	0.868	0.301	2.507
	More continuing education that is not sponsored by the pharmaceutical industry	−1.864	0.086	0.155	0.018	1.306

## Data Availability

The data used to support the findings of this study are available in the manuscript or its Supplementary Materials.

## Supplementary Materials

The following are available online at https://www.mdpi.com/article/10.3390/antibiotics11070867/s1, Supplementary File S1: Questionnaire on the knowledge, attitudes and behavior of veterinarians regarding antimicrobial use and antimicrobial resistance; Supplementary File S2: Dataset containing responses collected by questionnaire.
