# Supplementary material for: Veterinary Practitioners’ Standpoints and Comprehension towards Antimicrobial Use—Are There Opportunities for Antimicrobial Stewardship Improvement?
Source: Antibiotics (Basel). 2022 Jun 27;11(7):867. doi: 10.3390/antibiotics11070867 (PMC9311883; doi:10.3390/antibiotics11070867)
Supplement: Supplementary file 1 [file antibiotics-11-00867-s001.zip › Supplementary File S1.pdf]

antibiotics

MDPI

Supplementary File

Questionnaire on the Knowledge, Attitudes and Behavior of Veterinarians Regarding Antimicrobial Use and Antimicrobial Resistance

I Sociodemographic data

1. Age in years (write in): ______

2. Gender (circle): a) female
b) male

3. Do you work in (circle):
a) a state institution
b) a private institution

4. What year did you graduate from the Veterinary Faculty? (write in) _____

5. Your highest level of education (circle):
a) veterinary doctor
b) master of veterinary
c) doctor of veterinary medicine

5. Your highest level of education (circle):
a) doctor of medicine
b) master of medicine
c) doctor of medical Science

6. Your specialty (write in) _______________

7. Type of practice
   a) Companion animal only
   b) Equine or Bovine
   c) Mixed practice
   d) Other

8. Years in practice (circle): a) 0 - 5
b) 6 - 15
c) > 15

9. What number of patients are examined on average on a monthly basis? (write in): ______

II Significance of bacterial resistance to antibiotics in everyday work

1. Did you attend an education (seminar, educational gathering, continuous education) on the rational use of antibiotics or the antimicrobial resistance? (circle):

a) Yes, more than 3 years ago

b) Yes, over the last 3 years

c) I did not

d) I do not remember

2. How often do you encounter your everyday work with infection caused by bacteria resistant to most antibiotics? (circle):
daily / weekly / monthly / rare / never

3. What do you think, which sectors should be targeted to slower the development of antibiotic resistances? *Multiple selection*:

a) Hospital hygiene
b) Animal farm hygiene
c) Private food hygiene
d) AB use in hospitals
e) AB prescriptions by GP
f) AB intake by patients
g) AB use in livestock

III Prescribing behavior

1. Antibiotic prescribing changes compared to the similar period in the previous year (circle):
a) increased
b) decreased
c) no change

2. Specify 4 the most commonly prescribed antibiotics to your patients:

1. _____________
2. _____________
3. _____________
4. _____________

3. What are the reasons why antibiotics are prescribed without a hard indication? Multiple selection:

- when the weekend is approaching and the course of the disease is difficult to predict
- if the patient/pet owner demands an antibiotic
- if the patient/pet owner is incompliant
- language/cultural barriers when communicating with patients /pet owners
- lack of rapid diagnostic tests

- cost of microbiological testing
- lack of clear guidelines for some conditions

4. Do you routinely undertake any antibiograms in cases of therapeutic failure? Circle: Yes/No

5. How often do you prescribe the extra-label use of antimicrobial?
frequently/ moderately/ rarely or never

IV Information sources

1. Do you use practice guidelines domaće i strane for antibiotic therapy during your daily work? Circle:
frequently/ moderately/ rarely or never/ there are no good guidelines

2. Would you like to have more local guidelines for the rational use of antibiotics)
yes/ no/ don't know

3. Which are your sources to get current information on antibiotic therapy and ABR?
Multiple selection:
a) Internet forums
b) textbooks
c) scientific journals
d) clinical practice guidelines
e) direct communication with peer colleagues
f) direct communication with expert
g) continuing education

4. Which additional information sources would be particularly helpful?
Multiple selection:
a) no further sources needed, existing ones are sufficient
b) interdisciplinary network
c) clear national guidelines
d) more continuing education without industry sponsoring

V Knowledge on antimicrobial resistance

1. **On the scale shown, how much do you agree the following statements would help address the problem of antibiotic resistance? Single Code per statement**

		Agree Strongly	Agree Slightly	Neither agree nor disagree	Disagree Slightly	Disagree Strongly
**1.**	Antibiotic resistance is an important Public Health problem in our setting.	5	4	3	2	1

2.	The prescription of an antibiotic to a patient does not influence the possible appearance of resistance.	5	4	3	2	1
3.	I am convinced that new antibiotics will be developed to solve the problem of resistance.	5	4	3	2	1
4.	The use of antibiotics on animals is an important cause of the appearance of new resistance to pathogenic agents in humans.	5	4	3	2	1
5.	Two of the main causes of the appearance of antibiotic resistance are patient self-medication and antibiotic misuse.	5	4	3	2	1
6.	Dispensing antibiotics without a prescription should be more closely controlled.	5	4	3	2	1
7.	The phenomenon of resistance to antibiotics is mainly a problem in hospital settings.	5	4	3	2	1
